# Influence of Manual Double-Spin Preparation Protocols on the Cellular and Growth Factor Composition of Equine Platelet-Rich Plasma

**DOI:** 10.3390/ani16182824

**Published:** 2026-09-08

**Authors:** Andrea del Rincon, Michael J. S. Duggan, Milda Baltrimaite, Aoife O’Kane, John Mark O’Leary, Pieter A. J. Brama, Clodagh Kearney

**Affiliations:** School of Veterinary Medicine, University College Dublin, Belfield, D04 W6F6 Dublin, Ireland

**Keywords:** equine, platelet-rich plasma, orthobiologics

## Abstract

This prospective, randomised, ex vivo study investigated the effects of manual double-spin centrifugation protocols on the cellular and growth factor composition of equine platelet-rich plasma (PRP). Blood from 20 healthy horses was evaluated in two phases. In Phase A (n = 10), nine first-spin protocols were compared, and two were selected based on platelet enrichment and leukocyte reduction. In Phase B (n = 10), these were combined with nine second-spin conditions, generating 18 PRP preparations. Platelet (PLT), leukocyte (WBC) and erythrocyte (RBC) concentrations and PDGF-BB and TGF-β_1_ concentrations were measured. No significant differences in cellular composition were identified among first-spin protocols (*p* > 0.05). From Phase B, all final preparations achieved platelet enrichment and leukocyte depletion, with negligible RBC contamination. Second-spin force significantly affected PDGF-BB and TGF-β_1_ concentrations (*p* < 0.001). PDGF-BB increased from 2629 pg/mL (95% CI: 2111–3274) at 200× *g* to 3467 pg/mL (95% CI: 2784–4317) at 1000× *g*, while TGF-β_1_ increased from 13,364 pg/mL (95% CI: 11,286–15,824) to 16,998 pg/mL (95% CI: 14,355–20,127). TGF-β_1_ also increased with second-spin duration (*p* = 0.01). Platelet concentration was positively correlated with PDGF-BB (ρ = 0.604) and TGF-β_1_ (ρ = 0.691; both *p* < 0.001), whereas WBC concentration showed no significant association with either growth factor. Thus, both preparation methodology and donor-related variability influence equine PRP composition, indicating that protocol standardisation alone is unlikely to ensure biologically consistent PRP products.

## Simple Summary

Platelet-rich plasma is a blood product made by concentrating platelets, which are cells that release natural substances that can help repair damaged tissues. It is increasingly used in horses to treat injuries affecting tendons, ligaments, joints, and other tissues, but different preparation methods can produce products with very different compositions. This makes it difficult to compare reports and to know which method is most effective. The aim of this study was to compare a range of manual preparation methods and determine how they influenced the number of platelets, red and white blood cells, and growth factors in the final product. Blood samples from healthy horses were processed using different centrifugation methods and analysed in the laboratory. Although the preparation method influenced the final composition, differences between individual horses also had an effect. Horses naturally differed in the amount of healing proteins produced, even when identical methods were used. These findings show that improving preparation methods alone may not be enough to produce consistent platelet-rich plasma. Better characterisation of both the product and the individual donor could help improve the reliability of future treatments and support the development of more effective regenerative therapies for horses.

## 1. Introduction

Platelet-rich plasma (PRP) is a biological therapeutic product derived from whole blood after removal of red blood cells and partial or complete removal of white blood cells, resulting in a higher concentration of platelets within a reduced plasma volume [1,2,3,4]. Platelet-derived products (PDPs) obtained from whole blood have been explored as autologous or allogenic sources of growth factors (GFs), cytokines, and structural proteins with healing and regenerative potential [1].

PRP has increasingly been reported as a promising novel therapeutic in both human and veterinary medicine to treat a wide range of orthopaedic, soft tissue and dermatological conditions, and has recently gained attention for managing persistent breeding-induced endometritis (PBIE) in mares [5,6,7]. Based on positive outcomes in human medicine, including improvements in pain associated with known osteoarthritis [8,9,10], the use of PRP as an orthobiological product is now relatively widespread in veterinary medicine [4,11,12]. However, variable results in experimental equine studies have generated scepticism regarding its clinical effectiveness [2,13].

The immunomodulatory properties of PRP are attributed to growth factors and cytokines stored in the alpha granules of platelets [14]. For these proteins to be effective, platelet activation must occur. Platelet activation induces degranulation and the release of GFs, chemokines, and other bioactive molecules, including platelet-derived growth factor (PDGF), transforming growth factor-β_1_ (TGF-β_1_), vascular endothelial growth factor (VEGF), basic fibroblast growth factor (bFGF), platelet-derived epidermal growth factor (PDEGF), and insulin-like growth factors I and II (IGF-I/II) [15]. Activation is commonly induced by the addition of exogenous activators such as bovine thrombin or calcium chloride, while endogenous activation by exposure to stimuli in the environment they are exposed to is also reported [16].

PRP is typically prepared using a double-spin centrifugation technique [17]. During the first low-force centrifugation, whole blood separates into three layers: red blood cells, a buffy coat containing leukocytes, and platelet-containing plasma. The plasma fraction is extracted, and a second, higher-force centrifugation step is then used to concentrate the platelets within a reduced plasma volume [18]. Double-spin methods generally achieve greater platelet enrichment than single-spin techniques while reducing erythrocyte contamination [19,20,21]. The final composition of the PRP product depends on numerous methodological variables, including centrifugation force and duration, plasma collection technique, and whether the buffy coat is incorporated into the final preparation [22]. The inclusion of the buffy coat results in leukocyte-rich PRP, whereas its exclusion yields leukocyte-poor PRP. The biological significance of leukocytes remains controversial, with some authors advocating leukocyte depletion to reduce protease activity and oxidative stress, while others suggest that leukocytes contribute beneficial cytokines and antimicrobial properties [2,15].

Consequently, the term platelet-rich plasma encompasses a spectrum of biologically distinct products rather than a single, uniform therapeutic agent. Several classification systems have been proposed that categorise PRP according to platelet concentration, leukocyte content, activation status and preparation method [13]. However, none have achieved universal acceptance, and there remains no consensus regarding either the definition of PRP or the optimal method of preparation [2,23].

This lack of standardisation presents a major challenge when interpreting the published literature. Differences in preparation protocols—including manual, semi-automated and fully automated techniques produce products with markedly different cellular and growth factor compositions, which vary not only between methods but also between species and individual donors [24]. As a result, studies reporting the use of “PRP” may, in fact, be investigating biologically distinct products, making meaningful comparison of therapeutic outcomes difficult.

The problem is compounded by inconsistent methodological reporting. In a systematic review of 104 orthopaedic studies, Chahla et al. found that only 10% adequately described the PRP preparation protocol and only 16% reported the cellular composition of the final product [23]. These findings were echoed in a more recent systematic review specifically examining PRP for the treatment of equine joint disease, which found that only 29% of the included studies provided comprehensive reporting, highlighting the ongoing lack of methodological standardisation [17]. Without detailed reporting of preparation methods and PRP composition, it is impossible to determine whether differences in clinical outcomes reflect treatment efficacy or simply differences in the biological product administered. These findings emphasise the need for greater standardisation of PRP preparation and comprehensive characterisation of the final product. The systematic evaluation of manual double-spin protocols under controlled experimental conditions is lacking. Addressing this knowledge gap is essential to improve protocol standardisation, facilitate comparison between studies and ultimately optimise the biological consistency of PRP products.

The objective of the present study was to compare the cellular and growth factor composition of equine PRP generated using multiple manual double-spin centrifugation protocols. The authors hypothesised that increasing centrifugation forces and duration would result in greater platelet enrichment and consequently higher concentrations of PDGF-BB and TGF-β_1_.

## 2. Materials and Methods

### 2.1. Study Design

A prospective, randomised, ex vivo laboratory study was conducted from August 2024 to December 2025 at University College Dublin Veterinary Hospital. The study was approved by the Ethical Review Committee of University College Dublin (AREC-E-24-30) and followed institutional guidelines for the care and use of animals in research, and informed owner consent was obtained. Horses were eligible for inclusion if they were clinically healthy based on physical examination, were between 2 and 12 years of age and had no clinically relevant abnormalities on baseline haematology.

### 2.2. Signalment and Clinical Examination

Sex (mare, gelding, stallion), age (years), weight (kilograms), and baseline haematological values (PCV: packed cell volume, WBC: white blood cells, RBC: red blood cells, PLT: platelets) were recorded. All horses underwent thorough clinical examinations, which were unremarkable, and were hospitalised for elective procedures. According to the owners, none of the horses had a history of previous blood donation. An intravenous jugular catheter, long-stay catheter (14 G × 10 cm Milacath^®^; MILA International, Inc., Erlanger, KY, USA), with a male Luer-lock injection cap with a polyisoprene injection membrane (Vygon^®^; Vygon, Ecouen, France) was placed aseptically prior to the procedure. A baseline haematology was performed using the ProCyte DX Haematology Analyser (IDEXX^®^; IDEXX Laboratories, Inc., Westbrook, ME, USA) prior to blood collection.

### 2.3. Blood Collection

Blood was collected into a double blood bag (450 mL) containing CPDA anticoagulant, using a 16 G needle (Panvet^®^; Pan Veterinary, Kildare, Ireland) attached directly to the bung. Horses were sedated with an alpha-2 agonist and, in certain cases, opioids to facilitate concurrent clinical procedures. Blood was collected until the bag reached 500 mL, and the collection duration was recorded.

### 2.4. PRP Protocols

#### 2.4.1. Phase A

**Figure 1:**
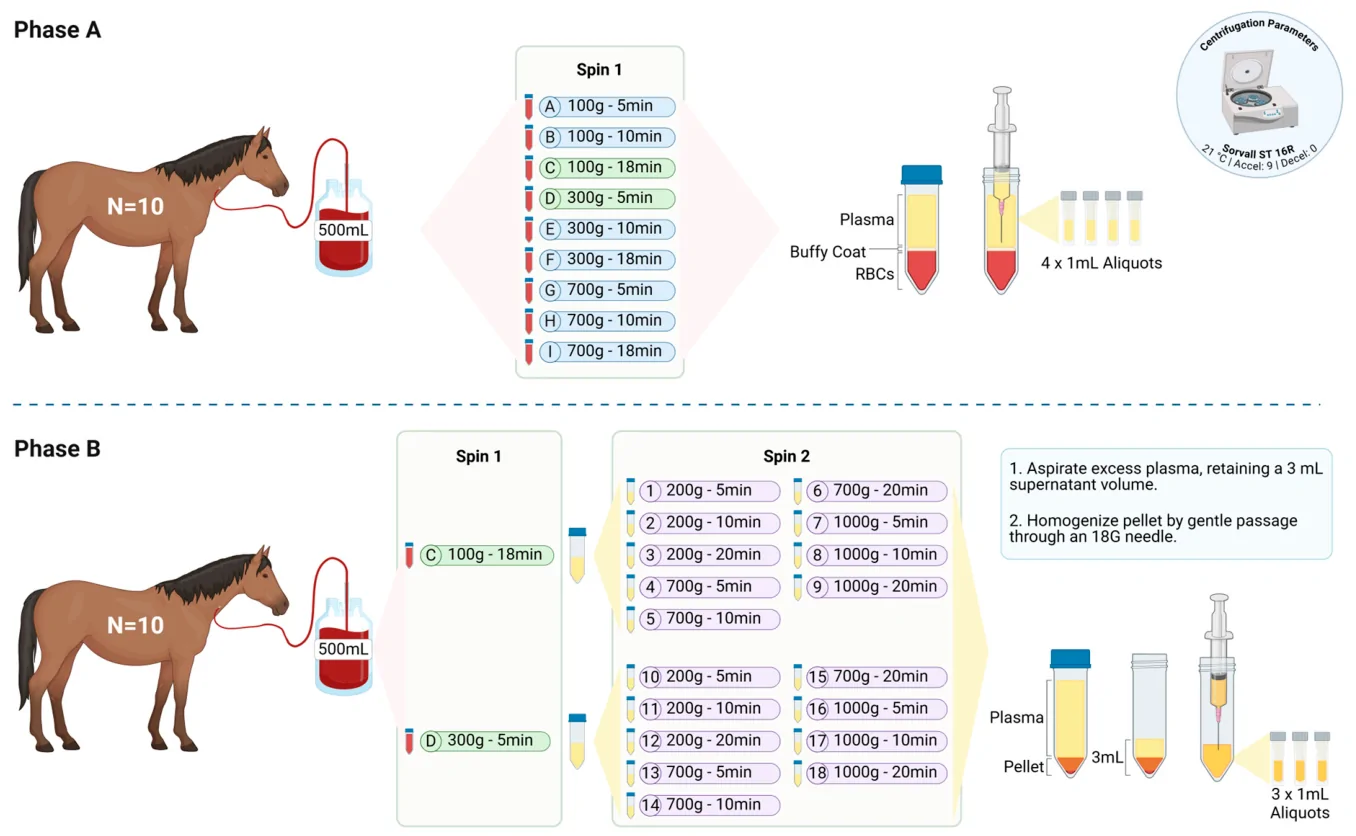
Schematic representation of Phase A and Phase B with the associated protocols. In Phase A, following each blood collection (n = 10) the blood was aliquoted into 9 × 50 mL conical tubes, and a single different (varying forces and durations) centrifuge cycle was performed on each tube. Following haematological analysis of the plasma obtained from each of these protocols, protocols C and D were selected to use in Phase B. In Phase B, blood was collected from 10 new horses. Half the blood from each horse underwent protocol C, the first-spin protocol from Phase A, and the other half underwent protocol D. Samples of plasma obtained following protocol C were centrifuged a second time, with each sample undergoing 1 of 9 different protocols (varying forces and duration). Different samples obtained using protocol D were also centrifuged using each of the 9 different Phase B protocols (from 10 to 18). On completion of the assigned double-spin protocol, the excess plasma was removed and only the last 3 mL of plasma and the platelet pellet were retained. This pellet was resuspended manually in the 3 mL of retained plasma and aliquoted in 3 × 1 mL samples. Created in BioRender. O’ Kane, A. (2026) https://BioRender.com/b3ht91l, accessed on 1 September 2026.

Following collection, the blood was transferred into 50 mL conical tubes (Thermo Fisher Scientific; Thermo Fisher Scientific, Waltham, MA, USA). Nine tubes per horse were centrifuged at different forces (100, 300, and 700 G) and durations (5, 10, and 18 min) (Figure 1). Centrifugation was performed using a Sorvall ST 16R centrifuge (Thermo Scientific) at 21 °C, with acceleration force set to 9 and deceleration force to 0. The same 9 protocols were performed for each of the 10 horses in a randomised order. Following centrifugation, 4 mL of plasma was aspirated a few millimetres above the buffy coat to minimise WBC contamination using a 20 mL syringe and an 18 G needle. Four 1 mL samples were aliquoted into 2 mL screw-cap tubes, and haematological analysis was performed on one sample from each protocol (Supplementary Table S1). The remaining samples were stored at −80 °C.

#### 2.4.2. Phase B

Following initial analysis of Phase A samples, two protocols were chosen based on WBC and PLT counts from Phase A. Phase B was conducted using a separate set of healthy horses (n = 10), using the same inclusion criteria. Blood collection and the first centrifugation were performed as previously described. A total of 200 mL of plasma from the first spin was obtained (100 mL from protocol C and 100 mL from protocol D), the samples were collected from just above the buffy coat and divided into 50 mL conical tubes (Thermo Fisher^®^). The samples were centrifuged for a second time at different forces (200, 700, and 1000× *g*) and durations (5, 10, and 20 min) in a randomised order (Figure 1). At the end of the second spin, the supernatant plasma in the conical tube was discarded from the upper portion, leaving only the 3 mL at the bottom and the platelet pellet. This platelet pellet was then resuspended in the retained 3 mL of plasma using a new 20 mL syringe fitted with an 18 G needle. The PRP was aspirated and mixed vigorously multiple times to ensure thorough resuspension (Figure 1 and Supplementary Table S1).

At the conclusion of Phase B, three samples (1 mL each) were aliquoted into 2 mL tubes. One tube was used for haematological analysis, and the remaining samples were stored at −82 °C.

### 2.5. Growth Factor Analysis

Both growth factors (PDGF-BB and TGF-β_1_) were chosen based on what was most commonly reported in the equine literature to allow for comparison [2,13,25,26]. Samples were stored at −80 °C prior to analysis, with storage durations ranging from 149 to 426 days for PDGF-BB and 128 to 388 days for TGF-β_1_. Previous work has demonstrated stability of PDGF-BB and TGF-β_1_ in equine PRP during frozen storage for up to six months [27]; however, some samples in the present study were stored for longer periods. Individual storage durations are provided in Supplementary Table S7. Growth factor ELISAs were performed following a single freeze–thaw cycle. Once thawed, all samples were gently mixed before analysis. ELISA plates were organised so that each plate contained all 18 PRP preparations from two horses, with each sample analysed in duplicate. All samples from an individual horse were therefore analysed on the same plate, and the same horse-to-plate allocation was used for both PDGF-BB and TGF-β_1_ assays. 

PDGF-BB concentrations were determined using a Quantikine^TM^ Human PDGF-BB sandwich enzyme immunoassay kit (R&D Systems, Bio-techne^®^, Minneapolis, MI, USA; Cat. No SBB00), as previously reported in equine PRP studies [13,18,19,20,21,24]. PDGF-BB samples were analysed in duplicate at a dilution of 1:20, and assays were conducted as per the manufacturer’s guidelines, except for the final step, where, on addition of the stop solution, the mixture was aspirated and dispensed five times to ensure homogeneity. The data were analysed using a computer software, generating a log/log curve fit.

TGF-B_1_ concentrations were determined using a Quantikine^TM^ Human TGF-B_1_ Human/Mouse/Rat/Porcine Canine sandwich enzyme immunoassay kit (R&D Systems, Bio-techne^®^, Minneapolis, MI, USA; Cat. No. SB100C), previously reported in equine PRP studies [13,18,19,20,21]. Prior to assaying, TGF-B_1_ samples were activated using a commercial Sample Activation Kit (R&D Systems^®^, Minneapolis, MN, USA; Cat. No. DY010); in summary, 40 μL of PRP was mixed with 10 μL of 12 N hydrochloric acid (HCl) in a polypropylene tube, homogenised and incubated at room temperature for 10 min. Thereafter, 10 μL of 10 N sodium hydroxide (NaOH) was added, followed by a further 10 min incubation period. Following activation, sample pH was verified at 7.2–7.6 using commercial pH strips (BMUT^®^ PRO; BMUT UG, 10117, Berlin, Germany; Lot. PHTS33S). TGF-β_1_ samples were analysed at a final dilution of 1:60, as per manufacturers guidelines. The data were analysed using a four-parameter logistic (4-PL) curve fit.

For both kits ([SB100C] and [SBB00]), optical density was measured within 30 min at a sample wavelength of 450 nm using a microplate reader (Absorbance 96; Byonoy GmbH, Hamburg, Germany) with a reference wavelength of 570 nm.

### 2.6. Statistical Analysis

In the first phase (A) the data were explored and normality was assessed using descriptive statistics (mean, median, skewness and kurtosis) and graphical assessment (histograms and Q-Q plots). A Friedman test was used to assess for statistically significant differences between Phase A protocols. In the second phase (B), generalised linear mixed-effects models (GLMMs), using a Gamma distribution with a log link function, were fitted with first-spin protocol (a combined first-spin force and spin time), second-spin force and second-spin time as fixed effects and horse as a random effect. Separate models were fit for each outcome variable (PDGF-BB, TGF-B_1_, PLT and WBC). The models included all main effects and two-way and three-way interactions among first-spin protocol, second-spin force and second-spin time. Model performance was assessed using marginal and conditional coefficients of determination (R^2^) [28]. Marginal R^2^ quantified the proportion of variance explained by fixed effects alone, whereas conditional R^2^ quantified the proportion of variance explained by both fixed and random (horse) effects. The effect of horse was approximated by the difference between R^2^_conditional_ and R^2^_marginal_.

Sample processing order was evaluated as a potential confounding variable in preliminary models and had no significant effect; therefore, it was not included in the final model. Model assumptions were assessed using simulation-based residual diagnostics (DHARMa), descriptive statistics (mean, median, skewness and kurtosis), graphical assessment (histograms, QQ plots, residual vs. fitted plots), and statistical tests (e.g., Shapiro–Wilk) where appropriate. Tukey was used for pairwise comparisons of estimated marginal means. Exploratory associations between PLT and WBC for PDGF-BB and TGF-β_1_ were assessed using Spearman’s rank correlation coefficient (*ρ*). Correlation coefficients and corresponding *p*-values were calculated for each variable pair. This analysis did not account for repeated observations within horses and as such the correlations were interpreted with caution. To investigate the relationship between platelet concentration and growth factor concentrations, mixed-effects generalised additive models (GAMs) were fitted for PDGF-BB and TGF-β_1_ with platelet concentration as the predictor and horse included as a random effect. Linear and nonlinear models were compared using Akaike’s Information Criterion (AIC) and effective degrees of freedom were used to assess the extent of nonlinearity. ΔAIC was calculated as the difference between the AIC of the linear model and AIC of the nonlinear model. Positive values favoured the nonlinear model, and negative values favoured the linear model.

All statistical tests were interpreted using a 5% level of significance (*p* < 0.05). All statistical analyses and plot generation were performed using R 4.6.0 statistical programming language (R Core, 2023). All data were included in the analysis unless specifically noted. The mean is reported with the standard deviation and median with 1st and 3rd quartile (Q1, Q3) where appropriate.

## 3. Results

Twenty healthy horses met the inclusion criteria (Phase A: n = 10; Phase B: n =10) with a median age of 4.5 years (IQR 4.5 years; range 2–11 years). The population consisted of nine mares, seven geldings, and four stallions of mixed breeds, with a mean body weight of 538.7 ± 59.6 kg.

### 3.1. Phase A

Considerable inter-horse variability was observed in PLT, WBC and RBC concentrations across the nine first-spin centrifugation protocols (Supplementary Table S2). No statistically significant differences were identified between protocols for any cellular variable (*p* > 0.05).

A ranking system was developed to organise and prioritise the protocols evaluated in Phase B based on their leukocyte and platelet concentrations. RBCs were not included in the ranking because their concentrations were close to zero across all preparations.

As the primary objective was to obtain leukocyte-poor PRP, the first criterion was the WBC concentration. Protocols with a WBC concentration below 2 × 10^9^/L were prioritised, with lower WBC counts receiving a higher ranking. Among protocols with similarly low WBC concentrations, platelet concentration was then used as the secondary criterion, with higher platelet counts receiving a higher ranking.

Based on the WBC criterion, the protocols were ranked as follows: D, C, E, F, G, H, I, A, B. Platelet concentration was then considered as the secondary criterion to differentiate between protocols with comparable WBC concentrations. The platelet ranking based on the haematology results was: B, A, D, C, E, G, F, H, I.

Using this hierarchical approach, prioritising leukocyte reduction while favouring higher platelet concentrations, protocols C and D were identified as the most suitable candidates for the final assessment in Phase B. This ranking system was therefore designed to reflect the intended characteristics of the final PRP product, rather than relying on platelet concentration alone (Supplementary Table S3).

### 3.2. Phase B

In Phase B, certain centrifugation protocols (6–7) were associated with increased PDGF-BB and TGF-β_1_ concentrations; however, no single protocol consistently outperformed all others across outcomes. Platelet concentrations were significantly increased compared with baseline blood across all samples (*p* < 0.001) whilst WBC concentrations were decreased (*p* < 0.001). Red blood cell contamination was negligible across all samples. Generalised linear mixed-effects models demonstrated that second-spin force significantly influenced both growth factors (*p* < 0.001), with higher centrifugation forces producing greater concentrations. Estimated marginal mean TGF-β_1_ concentrations increased with second-spin force, from 13,364 pg/mL (95% CI 11,286–15,824) at 200× *g* to 16,819 pg/mL (95% CI 14,204–19,915) at 700× *g* and 16,998 pg/mL (95% CI 14,355–20,127) at 1000× *g*.

Pairwise comparisons showed that both 700× *g* (*p* < 0.001) and 1000× *g* (*p* < 0.001) produced significantly higher TGF-β_1_ concentrations than 200× *g*, whereas 700× *g* and 1000× *g* did not differ significantly (*p* = 0.956). Similarly, PDGF-BB concentrations increased with second-spin force, from 2629 pg/mL (95% CI 2111–3274) at 200× *g* to 3139 pg/mL (95% CI 2521–3908) at 700× *g* and 3467 pg/mL (95% CI 2784–4317) at 1000× *g*. Pairwise comparisons showed that PDGF-BB concentrations were significantly higher at both 700× *g* (*p* < 0.001) and 1000× *g* (*p* < 0.001) than at 200× *g*, and were also significantly higher at 1000× *g* than at 700× *g* (*p* = 0.022). Second-spin time also significantly affected TGF-β_1_ concentration (*p* = 0.01). Estimated marginal mean TGF-β_1_ concentrations increased with second-spin time, from 14,834 pg/mL (95% CI 12,528–17,565) at 5 min to 15,121 pg/mL (95% CI 12,770–17,904) at 10 min and 17,033 pg/mL (95% CI 14,385–20,169) at 20 min. Pairwise comparisons showed that TGF-β_1_ concentrations were significantly higher at 20 min than at both 5 min (*p* < 0.001) and 10 min (*p* = 0.004), whereas no significant difference was observed between 5 and 10 min (*p* = 0.863). Significant two-way interactions were identified between second-spin force and time for PDGF-BB (*p* = 0.013) and TGF-β_1_ (*p* = 0.026), and three-way interactions between first-spin protocol, second-spin force and second-spin time for TGF-β_1_ (*p* = 0.031).

First-spin protocol, second-spin force and second-spin time did not significantly affect WBC concentrations (*p* = 0.473, *p* = 0.459, *p* = 0.9) or PLT concentrations (*p* = 0.724, *p* = 0.060, *p* = 0.245) overall, and no significant two-way or three-way interactions were detected. However, for PLT, although there was no overall effect of second-spin force, Tukey-adjusted pairwise comparisons showed that 700× *g* (*p* = 0.002) and 1000× *g* (*p* = 0.001) produced significantly higher PLT than 200× *g*.

Conditional R^2^ values were substantially higher than marginal R^2^ values for all mixed-effects models, indicating that between-horse variability accounted for a large proportion of the explained variance.

Spearman rank correlation analysis demonstrated strong positive correlations between PDGF-BB and PLT (ρ = 0.604, *p* < 0.001), and TGF-β_1_ and PLT (ρ = 0.691, *p* < 0.001). No significant correlations were observed between WBC and PDGF-BB (ρ = 0.121, *p* = 0.106) or WBC and TGF-β_1_ (ρ = −0.087, *p* = 0.245). Mixed-effects GAMs demonstrated a significant, positive association between platelet concentration and both growth factors. The PDGF-BB smooth exhibited minimal curvature (edf = 1.44), and the nonlinear model did not improve model fit relative to the linear model (ΔAIC = −0.03). In contrast, the nonlinear model was favoured for TGF-β_1_ (edf = 3.92; ΔAIC = 9.17), supporting a nonlinear association with platelet concentration.

All 18 PRP preparation protocols (Table 1) resulted in significant platelet enrichment relative to baseline whole blood (*p* < 0.001). Platelet concentrations varied significantly between protocols (*p* = 0.038), with an overall trend toward higher platelet concentrations across protocols 1–9, and 10–18. There was marked inter-horse variability (*p* < 0.001), with donor horse identity exerting a stronger influence on platelet concentration than protocol selection alone.

## 4. Discussion

This study evaluated the influence of multiple manual double-spin PRP centrifugation protocols on the cellular composition and growth factor content of equine PRP. Although the centrifugation protocol significantly affected platelet concentration and growth factor yield, donor-related biological variability also accounted for a proportion of the observed variation. Platelet concentration was the strongest predictor of PDGF-BB and TGF-β_1_ concentrations. Contrary to our hypothesis, increasing centrifugation force and duration did not consistently increase growth factor concentrations.

The absence of a significant increase in growth factor concentrations with higher centrifugation forces and longer spinning times may be explained by platelet structural damage (lysis) or premature activation under higher centrifugal forces, as well as a potential plateau effect in platelet recovery, which has been previously reported in human medicine [29]. In addition, inter-variability and methodological limitations in growth factor quantification may have further contributed to the lack of a dose-dependent response, as previously described by Jo C.H et al. in human medicine and by Textor et al. in equine veterinary medicine [13,30].

Platelet concentration has consistently been identified as a key predictor of the concentrations of these mediators (growth factors) because platelets represent the primary reservoir of α-granules—stored bioactive mediators [13,30]. Accordingly, our findings demonstrated that platelet concentration was the strongest predictor of PDGF-BB and TGF-β_1_ concentrations, supporting previous observations by Textor et al. and Giraldo et al., who also found positive associations between platelet concentration and growth factor release [13,25]. Nevertheless, caution is required when comparing these findings across studies because differences in PRP protocols, including both manual and automated methods, produce biologically distinct products that are not directly comparable [13,25]. In contrast to platelet concentration, leukocyte content were inversely proportional to growth factor concentrations. Although all protocols in the present study significantly reduced leukocyte concentrations relative to baseline whole blood, no significant differences in leukocyte concentration were observed between protocols, consistent with previous reports by Giraldo et al. [25] and Miranda et al. [3]. In contrast, Kazemi et al. reported positive associations between leukocyte concentration and both PDGF-BB and TGF-β_1_ concentrations, suggesting that leukocytes may contribute to the growth factor milieu through cytokine release or modulation of platelet activation [22]. We did not observe this relationship, which may reflect differences in study design, PRP preparation methodology or the relatively small sample size. Collectively, these findings support the concept that platelet concentration is the principal determinant of growth factor yield, whereas the biological contribution of leukocytes remains less clearly defined, and is likely to affect the intended clinical application [2,22,31,32,33,34].

An interesting finding of this study was the substantial variability associated with the horse-level random effect. This is consistent with intrinsic biological variability between donors being an important contributor to PRP composition, although a contribution of analytical variability cannot be completely excluded. Potential explanations for this variability include inter-individual differences in platelet count and activation capacity, variability in α-granule content and growth factor storage, genetic background, and systemic physiological factors such as age, sex, and hormonal or metabolic influences [13,29,34,35]. Similar donor-dependent variability has been widely reported in both human and equine PRP studies, supporting the concept that biological heterogeneity represents an inherent and potentially unavoidable limitation of autologous platelet-based therapies [13,25,35,36,37]. These findings further illustrate a major challenge for the standardisation of PRP products across individuals and species. Giraldo et al. reported an association between elevated PDGF-BB concentrations and young female horses; however, this could not be evaluated in the present study due to the limited sample size and population structure [25].

Protocol selection in Phase A was based on maximising platelet concentration while minimising leukocyte and erythrocyte contamination. This approach is supported by the aforementioned role of platelets as the primary source of growth factors involved in tissue repair, including PDGF-BB and TGF-β_1_ [29,38]. In contrast, leukocytes may contribute to inflammatory and catabolic effects through the release of proteases and reactive oxygen species, while erythrocyte contamination is associated with oxidative stress and a less favourable healing environment [39,40,41]. Although Phase A did not identify statistically significant differences in the nine initial centrifugation protocols, protocols C and D demonstrated favourable trends toward higher platelet recovery combined with lower leukocyte contamination. Protocol selection was based solely on platelet and leukocyte composition and therefore a potential for selection bias does exist and is a limitation of this study. The absence of statistically significant differences in Phase A may be attributable to the relatively small sample size and the substantial inter-individual variability observed among donor horses, which may have reduced the ability to detect subtle protocol-dependent effects.

In Phase B, certain centrifugation protocols (6–7) were associated with increased PDGF-BB and TGF-β_1_ concentrations; however, no single protocol consistently outperformed all others across outcomes. This variability highlights the interaction between centrifugation force, time, and the resultant cellular composition, as has previously been reported by Textor et al. [13].

PDGF-BB and TGF-β_1_ were chosen for investigation in this study due to their well-established central roles in regulating key regenerative processes, including chemotaxis, cellular proliferation, angiogenesis, and extracellular matrix synthesis, and their widespread study [13,42]. These processes are essential for tissue healing and repair, ultimately contributing to the restoration of normal tissue architecture and function [13]. Their consistent use as principal biomarkers in equine PRP research supports their suitability as accurate indicators of biologically relevant growth factor concentration across different preparation protocols [13,25,29].

In our study, all protocols were performed in a random order, with the total time from blood collection to the end of the last protocol being approximately 6 h. This did not result in any statistically significant effect on growth factor concentrations, which suggests that processing order was not a source of experimental bias. Moreover, the absence of significant changes in growth factor concentrations over this 6 h period may have potential clinical relevance, as it suggests that the timing of PRP processing within this timeframe may not substantially affect the resulting growth factor profile and is consistent with previous reports [14,43].

Although these manual methods may not be widely adopted, they could offer a cost-effective, simple, and accessible alternative for PRP preparation, while providing greater control over the resulting PRP composition. Similarly, scientific evidence demonstrating efficacy was the primary reason respondents chose to use biologic therapies, whereas the cost of these products was identified as the main limiting factor to their use [12,44].

This study has several limitations. The sample size was deemed appropriate for a controlled laboratory pilot study; it remains relatively small and may not fully capture the biological variability of the wider equine population. Consequently, some non-significant findings, particularly in Phase A, may reflect limited statistical power rather than the true absence of biologically meaningful differences. Growth factor assessment was limited to PDGF-BB and TGF-β_1_ using commercially available ELISA assays previously reported for equine PRP but not analytically validated specifically within the equine PRP matrix in this study. The concentrations reported represent growth factors detectable under the sample-processing conditions used and should not be interpreted as total or physiologically releasable growth factor content, which might not reflect in vivo biological activity or show the full spectrum of bioactive mediators.

Storage duration varied among horses, and some samples were stored for longer than the six-month period over which stability of PDGF-BB and TGF-β_1_ in equine PRP has previously been demonstrated [27]. An influence of prolonged or variable storage duration on measured growth factor concentrations therefore cannot be excluded. In addition, although all samples from an individual horse were analysed on the same ELISA plate, with each plate containing samples from two horses, each horse was represented on only one plate. This design minimised the potential influence of inter-plate variability on within-horse comparisons among PRP protocols; however, a contribution of inter-plate analytical variability to the variation associated with the horse-level random effect cannot be completely excluded.

Although absolute platelet and leukocyte concentrations were measured, overall product yield was not evaluated. The assessment of protocol performance was based primarily on the cellular composition of the resulting PRP. Although changes in platelet and leukocyte concentrations relative to baseline whole blood were evaluated, the volumes required to calculate total cellular recovery and overall product yield were not recorded. Consequently, formal platelet recovery efficiency and product yield could not be determined. This limits comparison of the overall efficiency of the different preparation protocols, as an increase in platelet concentration does not necessarily equate to greater recovery of the available platelets. Future studies should incorporate both cellular concentrations and volumetric measurements to allow assessment of platelet recovery, leukocyte reduction and overall product yield alongside growth factor composition. The absence of an appropriate baseline measurement for growth factor quantification represents a further potential limitation. Further investigation of baseline samples would therefore be beneficial for improving the accuracy and interpretation of the results and should be considered in future studies. A final limitation is that approximately 80% of the horses were sedated during blood collection. Sedation with α2-agonists has been reported to induce transient alterations in equine haematological parameters, particularly packed cell volume and erythrocyte distribution, mainly due to splenic sequestration and fluid shifts; however, available evidence suggests a minimal impact on platelet-derived growth factor content in PRP preparations under standardised processing conditions [45,46].

## 5. Conclusions

This study demonstrates that the composition of equine PRP is influenced by both the preparation protocol and substantial inter-individual variation between donor horses. Despite evaluating a wide range of manual double-spin centrifugation conditions, protocol-related effects accounted for only part of the observed variability in the final PRP products. Unsurprisingly, platelet concentration was strongly associated with PDGF-BB and TGF-β_1_ concentrations, while differences between individual horses also contributed substantially to variation in both cellular and growth factor composition.

These findings indicate that optimisation and standardisation of centrifugation protocols alone are unlikely to ensure consistent equine PRP products. Greater attention should therefore be given to characterisation of the final PRP product rather than assuming biological equivalence based on the preparation method used. Further investigation is warranted to identify the donor-related factors underlying the marked inter-individual variability observed and to determine whether these differences influence the biological activity and, ultimately, clinical efficacy of PRP.

## Figures and Tables

**Table 1:** Median (IQR) of the platelet (PLT), white blood cell (WBC), red blood cell (RBC), platelet-derived growth factor (PDGF-BB) and transforming growth factor-β_1_ (TGF-β_1_) concentration, grouped by first-spin protocol (C-100G during 18 min and D: 300G during 5 min).

**Group 1: First Spin Protocol C from Phase A: 100× *g* for 18** **min**										
**Protocol**	**Overall** **N = 90 ^1^**	**1** **N = 10 ^1^**	**2** **N = 10 ^1^**	**3** **N = 10 ^1^**	**4** **N = 10 ^1^**	**5** **N = 10 ^1^**	**6** **N = 10 ^1^**	**7** **N = 10 ^1^**	**8** **N = 10 ^1^**	**9** **N = 10 ^1^**
PLT	252 (179, 385)	189 (157, 211)	228 (199, 307)	222 (155, 363)	315 (190, 379)	269 (195, 496)	323 (185, 597)	314 (236, 599)	282 (217, 379)	277 (169, 475)
WBC	2.90 (1.95, 4.01)	2.68 (1.90, 3.59)	2.98 (2.16, 4.18)	2.57 (1.60, 4.17)	2.93 (2.43, 3.96)	2.87 (2.00, 4.35)	2.93 (1.82, 3.86)	3.42 (2.26, 4.07)	2.18 (1.88, 3.14)	2.97 (1.81, 4.01)
PDGF	2919 (2016, 4162)	2141 (1532, 2700)	2635 (1993, 3625)	2749 (2034, 4243)	3048 (1804, 4243)	3285 (2016, 4147)	2986 (2065, 4413)	3059 (2232, 5323)	3195 (2167, 5050)	3540 (2629, 4784)
TGF	15,254 (12,303, 19,141)	10,737 (9253, 14,559)	12,226 (11,477, 14,248)	15,544 (13,723, 16,982)	16,986 (11,885, 20,622)	15,118 (12,589, 23,402)	15,966 (12,419, 25,405)	16,385 (14,268, 22,544)	15,460 (12,373, 22,197)	17,403 (13,466, 20,806)
**Group 2: First Spin Protocol D from Phase A: 300× *g* for 5** **min**										
**Protocol**	**Overall** **N = 90 ^1^**	**10** **N = 10 ^1^**	**11** **N = 10 ^1^**	**12** **N = 10 ^1^**	**13** **N = 10 ^1^**	**14** **N = 10 ^1^**	**15** **N = 10 ^1^**	**16** **N = 10 ^1^**	**17** **N = 10 ^1^**	**18** **N = 10 ^1^**
PLT	273 (180, 441)	175 (140, 215)	270 (171, 294)	214 (154, 308)	305 (211, 441)	303 (245, 518)	284 (154, 468)	236 (182, 544)	299 (218, 461)	336 (178, 445)
WBC	3.31 (2.31, 5.30)	3.37 (1.54, 5.69)	3.25 (1.90, 5.56)	3.32 (2.28, 5.21)	3.79 (2.58, 5.30)	2.77 (2.41, 3.96)	3.21 (2.21, 4.24)	3.25 (2.63, 7.61)	3.00 (2.31, 6.12)	3.75 (2.48, 5.29)
PDGF	3047 (2330, 4353)	2608 (1672, 3139)	2624 (2441, 3601)	2704 (2101, 3716)	3279 (2442, 4885)	3298 (2356, 4462)	2996 (2057, 5116)	3066 (2562, 4551)	3494 (1844, 3951)	4069 (3073, 5296)
TGF	15,613 (11,972, 19,200)	11,134 (9224, 13,229)	13,936 (11,140, 16,352)	14,353 (10,947, 16,509)	17,154 (15,213, 17,972)	15,696 (12,233, 21,780)	15,322 (13,352, 22,469)	17,605 (11,357, 19,200)	15,996 (11,690, 19,641)	18,951 (15,934, 20,685)

^1^ Median (Q1, Q3).

## Data Availability

The data have restricted access due to ethical considerations but are available upon request from the authors.

## Supplementary Materials

The following supporting information can be downloaded at https://www.mdpi.com/article/10.3390/ani16182824/s1. Figure S1: the plate layout; Table S1: Summary of the Phase A and Phase B protocol characteristics (force and duration), final volume, and resuspension method; Table S2: Phase A median (IQR) platelet (PLT), white blood cell (WBC), and red blood cell (RBC) concentrations at baseline for the 10 horses used in Phase A. The overall baseline blood average is presented at the end; Table S3: Phase A median (IQR) platelet (PLT), white blood cell (WBC), and red blood cell (RBC) concentrations for each first-spin protocol along with the baseline. Protocols C and D were selected for further evaluation in Phase B; Table S4: Phase B median (IQR) platelet (PLT), white blood cell (WBC), and red blood cell (RBC) concentrations at baseline for the 10 horses used in Phase B; Table S5: Median (IQR) of the platelet (PLT), white blood cell (WBC), red blood cell (RBC), platelet-derived growth factor (PDGF-BB) and transforming growth factor-β_1_ (TGF-β_1_) concentration, grouped by first spin protocol (C: 100 G during 18 min and D: 300 G during 5 min); Table S6: Enrichment or depletion of WBC, PLT, and RBC in the 18 final PRP preparations from the 10 horses included in Phase B. The ratio was calculated by dividing the value obtained for each protocol by the baseline value for the same horse. All values were rounded to two decimal; Table S7: ELISA plate allocation and storage duration of Phase B samples prior to growth-factor analysis.
