# Supplementary material for: Influence of Manual Double-Spin Preparation Protocols on the Cellular and Growth Factor Composition of Equine Platelet-Rich Plasma
Source: Animals (Basel). 2026 Sep 8;16(18):2824. doi: 10.3390/ani16182824 (PMC13603841; doi:10.3390/ani16182824)
Supplement: Supplementary file 1 [file animals-16-02824-s001.zip › animals-4478716-Supplementary.pdf]

PROTOCOLS PHASE A	FORCES	TIMING	FINAL PRP VOLUME	RESUSPENSION PROCEDURES
Protocol A	100G	5 minutes	4 * 1 ml	Resuspension with syringe and needle (18G)
Protocol B	300G	10 minutes	4 * 1 ml	
Protocol C	700G	18 minutes	4 * 1 ml	
Protocol D	100G	5 minutes	4 * 1 ml	
Protocol E	300G	10 minutes	4 * 1 ml	
Protocol F	700G	18 minutes	4 * 1 ml	
Protocol G	100G	5 minutes	4 * 1 ml	
Protocol H	300G	10 minutes	4 * 1 ml	
Protocol I	700G	18 minutes	4 * 1 ml	
**PROTOCOLS PHASE B**				
Protocol 1 & 10	200G	5 minutes	3 * 1 ml	Resuspension with syringe and needle (18G)
Protocol 2 & 11	700G	10 minutes	3 * 1 ml	
Protocol 3 & 12	1000G	20 minutes	3 * 1 ml	
Protocol 4 & 13	200G	5 minutes	3 * 1 ml	
Protocol 5 & 14	700G	10 minutes	3 * 1 ml	
Protocol 6 & 15	1000G	20 minutes	3 * 1 ml	
Protocol 7 & 16	200G	5 minutes	3 * 1 ml	
Protocol 8 & 17	700G	10 minutes	3 * 1 ml	
Protocol 9 & 18	1000G	20 minutes	3 * 1 ml	

Supplementary Table S1: Summary of the Phase A and Phase B protocol characteristics (force and duration), final volume, and resuspension method.

Baseline Blood	Baseline blood Horse 1	Baseline blood Horse 2	Baseline blood Horse 3	Baseline blood Horse 4	Baseline blood Horse 5	Baseline blood Horse 6	Baseline blood Horse 7	Baseline blood Horse 8	Baseline blood Horse 9	Baseline blood Horse 10	Baseline blood (average)
PLT: Median (IQR)	93 K/uL	93 K/uL	175 K/uL	135 K/uL	68 K/uL	83 K/uL	75 K/uL	130 K/uL	119K/uL	103K/uL	93 (83–130)K/uL
WBC: Median (IQR)	6.09 x 10'9/L	6.09 x 10'9/L	11.04 x 10'9/L	6.89 x 10'9/L	9.17x 10'9/L	6.57x10'9/L	5.38x10'9/L	4.38x10'9/L	9.11 X 10'9x10'9/L	6.63 X 10'9/L	8.00 (5.74–10.68) X 10'9/L
RBC: Median (IQR)	5.46 x x10'9/L	5.46 x x10'9/L	7.83 x10'9/L	6.53x10 '9/L	7.97x10'9/L	6.57x10'9/L	12.85x10'9/L	5.48x10'9/L	8.18x10'9	7.68 X 10'9/L	7.76 (6.53–8.18) X 10'9/L

<u>Supplementary Table S2</u>: Phase A median (IQR) platelet (PLT), white blood cell (WBC), and red blood cell (RBC) concentrations at baseline for the 10 horses used in Phase A. The overall baseline blood average is presented at the end.

Protocol	A (PRP)	B (PRP)	C (PRP)	D (PRP)	E (PRP)	F (PRP)	G (PRP)	H (PRP)	I (PRP)
PLT: Median (IQR)	141.0 (127.5–157.5) K/uL	142.0 (131.3–153.8) K/uL	124.5 (110.8–142.0) K/uL	139.5 (112.8–159.0) K/uL	111.0 (101.8–124.8) K/uL	77.0 (69.0–97.3) K/uL	99.5 (94.3–115.0) K/uL	58.5 (52.3–84.3) K/uL	30.0 (18.5–39.8) K/uL
WBC: Median (IQR)	7.37 (6.35–9.63) X 10'9/L	2.75 (2.52–3.18) X 10'9/L	0.93 (0.67–1.11) X 10'9/L	1.31 (0.61–1.50) X 10'9/L	0.12 (0.09–0.34) X 10'9/L	0.05 (0.01–0.14) X 10'9/L	0.04 (0.02–0.04) X 10'9/L	0.01 (0–0.02) X 10'9/L	0.01 (0–0.02) X 10'9/L
RBC: Median (IQR)	0.02 (0.01–0.04) X 10'9/L	0.01 (0.01–0.01) X 10'9/L	0.00 (0–0.00) X 10'9/L	0.00 (0–0.01) X 10'9/L	0.00 (0–0.02) X 10'9/L	0.00 (0–0.00) X 10'9/L	0.00 (0–0.00) X 10'9/L	0.00 (0–0.01) X 10'9/L	0.00 (0–0.00) X 10'9/L

Supplementary Table S3: Phase A median (IQR) platelet (PLT), white blood cell (WBC), and red blood cell (RBC) concentrations for each first-spin protocol along with the baseline. Protocols C and D were selected for further evaluation in Phase B.

Haematology	Horse 1	Horse 2	Horse 3	Horse 4	Horse 5	Horse 6	Horse 7	Horse 8	Horse 9	Horse 10
WBC (x 10'9/L)	6.66	7.8	9.42	9.51	7.76	7.91	7.67	6.89	8.49	9.02
RBC (x 10'9/L)	6.32	7.35	8.23	8.08	7.55	7.82	6.73	9.13	6.73	13.91
PLT (K/uL)	113	105	90	106	145	41	139	36	130	124

Supplementary Table S4: Phase B median (IQR) platelet (PLT), white blood cell (WBC), and red blood cell (RBC) concentrations at baseline for the 10 horses used in Phase B.

First Spin Speed Group 100G for 18 min										
Protocol	1 N=10[1]	2 N=10[1]	3 N=10[1]	4 N=10[1]	5 N=10[1]	6 N=10[1]	7 N=10[1]	8 N=10[1]	9 N=10[1]	Overall
PLT (K/uL)	189 (199-307)	228 (199-307)	222 (155-363)	315 (190-379)	269 (195-496)	323 (185-597)	314 (236-599)	282 (217-379)	277 (169-475)	252 (179-385)
WBC (x 10'9/L)	2.68 (1.9-3.59)	2.98 (2.16-4.18)	2.57 (1.60-4.17)	2.93 (2.43-3.96)	2.87 (2.00-4.35)	2.93 (1.82-3.86)	3.42 (2.26-4.07	2.18 (1.88-3.14)	2.97 (1.81-4.01)	2.90 (1.95-4.01)
PDGF-BB (pg/mL)	2.14 (1.53-2.7)	2.63 (1.99-3.62)	2.74 (2.03-4.24)	3.04 (1.8-4.24)	3.25 (2.01-4.14)	2.98 (2.06-4.41)	3.06 (2.23-5.32)	3.19 (2.17-5.05)	3.54 (2.62-4.78)	2.91 (2.01-4.15)
TGF-B_1(pg/mL)	10.7 (9.25-14.55)	12.22 (11.47-14.24)	15.54 (13.72, 16.98)	16.98 (11.88-20.62)	15.118 (12.59-23.41)	15.97 (12.42-24.41)	16.38 (14.26-22.54)	15.46 (12.37-22.19)	17.40 (13.46-20.81)	15.24 (12.30-19.14)
First Spin Speed Group 300G for 5 min										
Protocol	10 N=10[1]	11 N=10[1]	12 N=10[1]	13 N=10[1]	14 N=10[1]	15 N=10[1]	16 N=10[1]	17 N=10[1]	18 N=10[1]	Overall
PLT (K/uL)	175 (140-215)	270 (171-294)	214 (154-308)	305 (211-441)	303 (245-518)	284 (154-468)	236 (182-544)	299 (218-461)	336 (178-445)	272 (180-411)
WBC (x 10'9/L)	3.37 (1.54-5.69)	3.25 (1.90-5.56)	3.32 (2.28-5.21)	3.79 (2.58-5.30)	2.77 (2.41-3.96)	3.21 (2.21-4.24)	3.25 (2.63-7.61)	3.00 (2.31-6.12)	3.75 (2.48-5.29)	3.31 (2.31-5.30)
PDGF-BB (pg/mL)	2.60 (1.67-3.13)	2.62 (2.44-3.60)	2.70 (2.10-3.71)	3.27 (2.44-4.88)	3.29 (2.35-4.46)	2.99 (2.05-5.11)	3.07 (2.56-4.55)	3.49 (1.844-39.51)	4.07 (3.07-5.29)	3.04 (2.33-435)
TGF-B_1(pg/mL)	11.13 (9.22-13.22)	13.93 (11.14-16.35)	14.35(10.94-16.50)	17.15 (15.21-17.97)	15.69 (12.23-21.78)	15.32 (13.35-22.46)	17.60 (11.36-19.20)	15.99 (11.69-19.64)	18.95 (15.94-20.69)	15.61 (11.97-19.20)
[1] Median (Q1,Q3)										

Supplementary Table S5: Median (IQR) of the platelet (PLT), white blood cell (WBC), red blood cell (RBC), platelet-derived growth factor (PDGF-BB) and transforming growth factor-β_1 (TGF-β_1) concentration, grouped by first spin protocol (C: 100G during 18min and D: 300G during 5 min).

Protocol		1	2	3	4	5	6	7	8	9	10	11	12	13	14	15	16	17	18
**Enrichment or Depletion- Horse 1**	**WBC**	x0.4	x0.45	x0.39	x0.44	x0.43	x0.44	x0.51	x0.33	x0.45	x0.51	x0.4	x0.5	x0.57	x0.42	x0.48	x0.49	x0.45	x0.56
	**PLT**	x1.67	x2.02	x1.96	x2.79	x2.38	x2.86	x2.78	x2.5	x2.45	x1.55	x2.39	x1.89	x2.7	x2.68	x2.51	x2.09	x2.65	x2.97
	**RBC**	0	0	0	0	0	0	0	0	0	0	0	0	0	0	0	0	0	0
**Enrichment or Depletion- Horse 2**	**WBC**	x0.34	x0.38	x0.33	x0.38	x0.37	x0.38	x0.44	x0.28	x0.38	x0.43	x0.42	x0.43	x0.49	x0.36	x0.41	x0.42	x0.38	x0.48
	**PLT**	x1.8	x2.17	x2.11	x3	x2.56	x3.08	x2.99	x2.69	x2.64	x1.67	x2.57	x2.04	x2.9	x2.89	x2.7	x2.25	x2.85	x3.2
	**RBC**	0	0	0	0	0	0	0	0	0	0	0	0	0	0	0	0	0	0
**Enrichment or Depletion- Horse 3**	**WBC**	x0.28	x0.32	x0.27	x0.31	x0.3	x0.31	x0.36	x0.23	x0.32	x0.36	x0.35	x0.35	x0.4	x0.29	x0.34	x0.35	x0.32	x0.4
	**PLT**	x2.1	x2.53	x2.47	x3.5	x2.99	x3.59	x3.49	x3.13	x3.08	x1.94	x3	x2.38	x3.39	x3.37	x3.16	x2.62	x3.32	x3.73
	**RBC**	0	0	0	0	0	0	0	0	0	0	0	0	0	0	0	0	0	0
**Enrichment or Depletion- Horse 4**	**WBC**	x0.28	x0.31	x0.27	x0.31	x0.3	x0.31	x0.36	x0.23	x0.31	x0.35	x0.34	x0.35	x0.4	x0.29	x0.34	x0.34	x0.32	x0.39
	**PLT**	x1.78	x2.15	x2.09	x2.97	x2.54	x3.05	x2.96	x2.66	x2.61	x1.65	x2.55	x2.02	x2.88	x2.86	x2.68	x2.23	x2.82	x3.17
	**RBC**	0	0	0	0	0	0	0	0	0	0	0	0	0	0	0	0	0	0
**Enrichment or Depletion- Horse 5**	**WBC**	x0.35	x0.38	x0.33	x0.38	x0.37	x0.38	x0.44	x0.28	x0.38	x0.43	x0.42	x0.43	x0.49	x0.36	x0.41	x0.42	x0.39	x0.48
	**PLT**	x1.3	x1.57	x1.53	x2.17	x1.86	x2.23	x2.17	x1.94	x1.91	x1.21	x1.86	x1.48	x2.1	x2.09	x1.96	x1.63	x2.06	x2.32
	**RBC**	0	0	0	0	0	0	0	0	0	0	0	0	0	0	0	0	0	0
**Enrichment or Depletion- Horse 6**	**WBC**	x0.34	x0.38	x0.32	x0.37	x0.36	x0.37	x0.43	x0.28	x0.38	x0.43	x0.41	x0.42	x0.48	x0.35	x0.41	x0.41	x0.38	x0.47
	**PLT**	x4.61	x5.56	x5.41	x7.68	x6.56	x7.88	x7.66	x6.88	x6.76	x4.27	x6.59	x5.22	x7.44	x7.39	x6.93	x5.76	x7.29	x8.2
	**0**	0	0	0	0	0	0	0	0	0	0	0	0	0	0	0	0	0	0

**Enrichment or Depletion- Horse 7**	**WBC**	x0.35	x0.39	x0.34	x0.38	x0.37	x0.38	x0.45	x0.28	x0.39	x0.44	x0.42	x0.43	x0.49	x0.36	x0.42	x0.42	x0.39	x0.49
	**PLT**	x1.36	x1.64	x1.6	x2.27	x1.94	x2.32	x2.26	x2.03	x1.99	x1.26	x1.94	x1.54	x2.19	x2.18	x2.04	x1.7	x2.15	x2.42
	**RBC**	0	0	0	0	0	0	0	0	0	0	0	0	0	0	0	0	0	0
**Enrichment or Depletion- Horse 8**	**WBC**	x0.35	x0.39	x0.34	x0.38	x0.37	x0.38	x0.45	x0.28	x0.39	x0.44	x0.42	x0.43	x0.49	x0.36	x0.42	x0.42	x0.39	x0.49
	**PLT**	x5.25	x6.33	x6.17	x8.75	x7.47	x8.97	x8.72	x7.83	x7.69	x4.86	x7.5	x5.94	x8.47	x8.42	x7.89	x6.56	x8.31	x9.33
	**RBC**	0	0	0	0	0	0	0	0	0	0	0	0	0	0	0	0	0	0
**Enrichment or Depletion- Horse 9**	**WBC**	x0.32	x0.35	x0.3	x0.35	x0.34	x0.35	x0.4	x0.26	x0.35	x0.4	x0.38	x0.39	x0.45	x0.33	x0.38	x0.38	x0.35	x0.44
	**PLT**	x1.45	x1.75	x1.71	x2.42	x2.07	x2.48	x2.42	x2.17	x2.13	x1.35	x2.08	x1.65	x2.35	x2.33	x2.18	x1.82	x2.3	x2.58
	**RBC**	0	0	0	0	0	0	0	0	0	0	0	0	0	0	0	0	0	0
**Enrichment or Depletion- Horse 10**	**WBC**	x0.3	x0.33	x0.28	x0.32	x0.32	x0.32	x0.38	x0.24	x0.33	x0.37	x0.36	x0.37	x0.42	x0.31	x0.36	x0.36	x0.33	x0.42
	**PLT**	x1.52	x1.84	x1.79	x2.54	x2.17	x2.6	x2.53	x2.27	x2.23	x1.41	x2.18	x1.73	x2.46	x2.44	x2.29	x1.9	x2.41	x2.71
	**RBC**	0	0	0	0	0	0	0	0	0	0	0	0	0	0	0	0	0	0

Supplementary Table S6: Enrichment or depletion of WBC, PLT, and RBC in the 18 final PRP preparations from the 10 horses included in Phase B. The ratio was calculated by dividing the value obtained for each protocol by the baseline value for the same horse. All values were rounded to two decimal.[7]

7. Carmona, J.U.; Hernández-Valencia, J.D.; López, C. Molecular and Regenerative Effects of Platelet-Rich Plasma and Related Hemocomponents in Animal Models of Liver Injury—A Systematic Review. *Int. J. Mol. Sci.* **2026**, *27*, 1013, doi:10.3390/ijms27021013.

Horse Number	ELISA Plate	PRP Protocol	Replicates	Storage duration before PDGF-BB (days)	Storage duration before TGF-B$_1$ (days)
1	1	1 to 18	Duplicate	426	388
2	1	1 to 18	Duplicate	426	388
3	2	1 to 18	Duplicate	395	378
4	2	1 to 18	Duplicate	262	245
5	3	1 to 18	Duplicate	182	162
6	3	1 to 18	Duplicate	174	154
7	4	1 to 18	Duplicate	183	163
8	4	1 to 18	Duplicate	181	161
9	5	1 to 18	Duplicate	183	162
10	5	1 to 18	Duplicate	149	128

Supplementary Table S7: ELISA plate allocation and storage duration of Phase B samples prior to growth-factor analysis. All 18 PRP preparations from each horse were analysed on the same ELISA plate, and each sample was analysed in duplicate. Each plate contained samples from two horses. The same horse-to-plate allocation was used for the PDGF-BB and TGF-β1 assays. Storage duration represents the interval between sample collection/freezing and ELISA analysis. **Supplemental Figure S1** (below) illustrates the plate layout used.

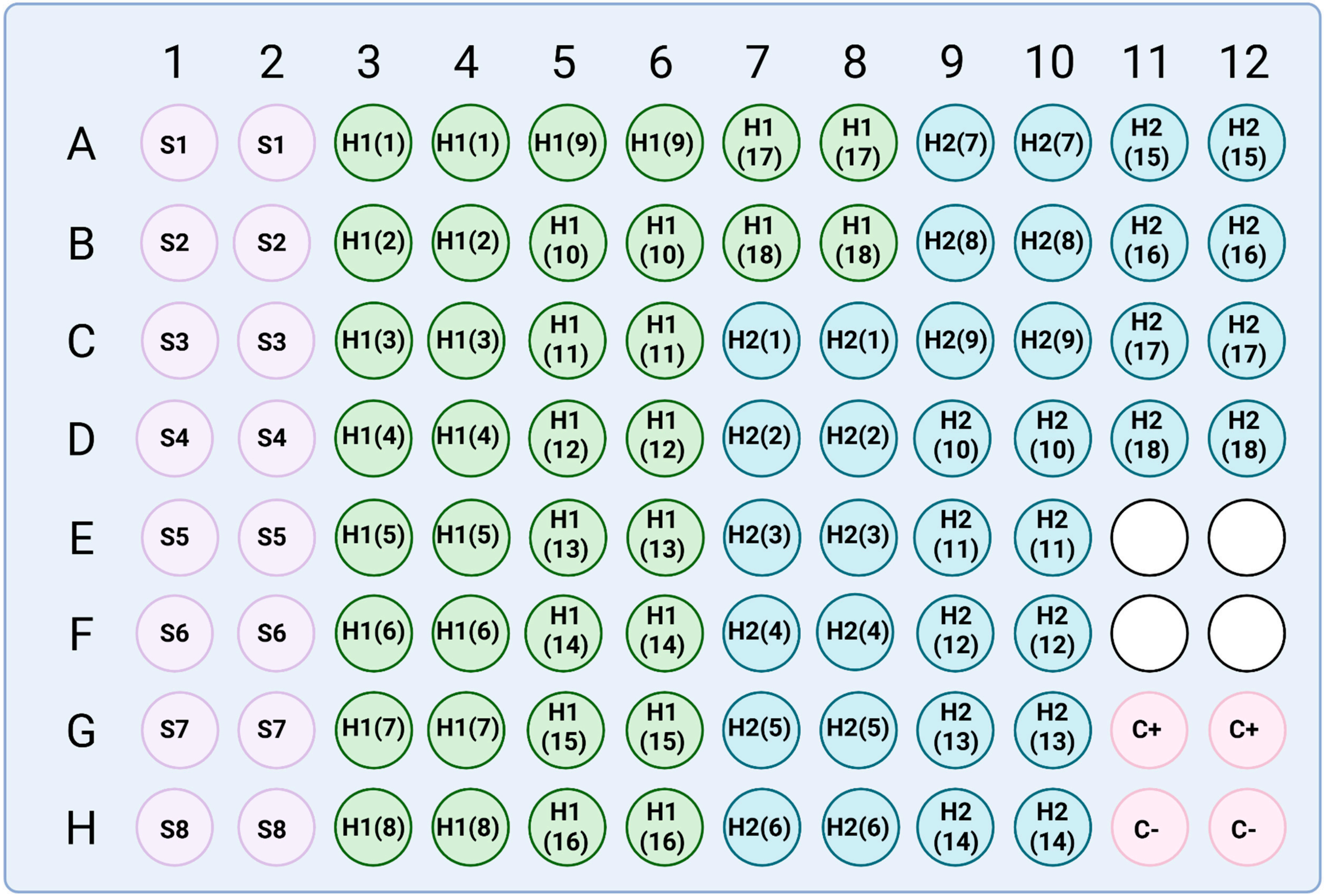

S = Standard H#(#) = Horse ID(Sample Number) C+ = Positive Control C- = Negative Control
